# Cellulose and hemicellulose decomposition by forest soil bacteria proceeds by the action of structurally variable enzymatic systems

**DOI:** 10.1038/srep25279

**Published:** 2016-04-29

**Authors:** Rubén López-Mondéjar, Daniela Zühlke, Dörte Becher, Katharina Riedel, Petr Baldrian

**Affiliations:** 1 Laboratory of Environmental Microbiology, Institute of Microbiology of the CAS, Vestec, 25242, Czech Republic; 2 Institute of Microbiology, Ernst-Moritz-Arndt-University of Greifswald, Greifswald, D-17487, Germany

## Abstract

Evidence shows that bacteria contribute actively to the decomposition of cellulose and hemicellulose in forest soil; however, their role in this process is still unclear. Here we performed the screening and identification of bacteria showing potential cellulolytic activity from litter and organic soil of a temperate oak forest. The genomes of three cellulolytic isolates previously described as abundant in this ecosystem were sequenced and their proteomes were characterized during the growth on plant biomass and on microcrystalline cellulose. *Pedobacter* and *Mucilaginibacter* showed complex enzymatic systems containing highly diverse carbohydrate-active enzymes for the degradation of cellulose and hemicellulose, which were functionally redundant for endoglucanases, β-glucosidases, endoxylanases, β-xylosidases, mannosidases and carbohydrate-binding modules. *Luteibacter* did not express any glycosyl hydrolases traditionally recognized as cellulases. Instead, cellulose decomposition was likely performed by an expressed GH23 family protein containing a cellulose-binding domain. Interestingly, the presence of plant lignocellulose as well as crystalline cellulose both trigger the production of a wide set of hydrolytic proteins including cellulases, hemicellulases and other glycosyl hydrolases. Our findings highlight the extensive and unexplored structural diversity of enzymatic systems in cellulolytic soil bacteria and indicate the roles of multiple abundant bacterial taxa in the decomposition of cellulose and other plant polysaccharides.

Forest soils are among the most important terrestrial carbon sinks covering vast areas, especially in the Northern hemisphere. The bulk of the plant primary production in these ecosystems eventually becomes part of the dead plant biomass in the form of the aboveground and belowground litter, mostly leaf litter^1^. Because most labile compounds, such as soluble oligosaccharides, organic acids or amino acids, are withdrawn by the trees during leaf senescence, dead plant biomass is composed mostly of cellulose and hemicelluloses accompanied by other biopolymers such as lignin and pectin^2^. For this reason, cellulose and hemicelluloses represent the main types of complex carbon compounds entering the soil environment, and their degradation represents a key step in the terrestrial carbon cycle^3^. Moreover, the transformation of cellulose and hemicelluloses is also of great interest for biotechnology due to the potential to deliver value-added products, such as bioethanol, and sustainable energy from biomass and complex agricultural wastes^4^.

The most important enzymes involved in the decomposition of cellulose in the dead plant biomass, cellulases, cleave the β-1,4 bond in the cellulose chain. In a classical view, cellulases are catalogued according to their mechanisms of action as endocellulases (cleaving internal bonds in the cellulose chain), exocellulases or cellobiohydrolases (acting on the reducing or non-reducing ends of cellulose chains) and β-glucosidases (converting cellobiose into glucose molecules)^5^,^6^. Cellulases belong to the carbohydrate-active enzymes (CAZymes), which are enzymes with catalytic and carbohydrate-binding modules (or functional domains) that degrade, modify, or create glycosidic bonds (http://www.cazy.org/)^7^. Most enzymes with cellulolytic activity are hydrolases (glycoside hydrolases, GHs), which have been classified into different families according to their amino acid sequence similarities and their resulting protein structures. Enzymes with characterized cellulolytic activity belong mainly to the families GH1, GH3, GH5, GH6, GH8, GH9, GH12, GH45, GH48, GH51 and GH74^8^. Even if the proteins within each family share structural properties, they have different evolutionary histories and do not always share substrate specificities. Many GH families include enzymes that can act on multiple substrates^9^. Similar to cellulases, hemicellulases are responsible for the degradation of hemicelluloses, such as xylans, xyloglucans, arabinoxylans and glucomannans, from plant biomass^10^. Again, there is an important difference between endo- and exo-cleaving enzymes, but the complexity of hemicellulose composition typically requires the action of a wide set of enzymes such as the endoxylanases, endomannanases, xylosidases, glucosidases, arabinosidases, galactosidases, mannosidases and glucuronidases. Enzymes with hemicellulolytic activities are mainly classified into the GH2, GH10, GH11, GH16, GH26, GH30, GH31, GH39, GH42, GH43 and GH53 families. Again, because members of some GH families catalyse different reactions, their family membership may not sufficiently imply the targets of their activity. This can be most reliably achieved by the comparison of protein sequences with biochemically characterized CAZymes^8^. In addition to the catalytic hydrolytic domain, cellulases and hemicellulases may also contain carbohydrate binding modules (CBM) that bind cellulose or hemicelluloses and can be classified into the CAZy families CBM1, CBM2, CBM3, CBM6, CBM8, CBM30 and CBM44, among others. Together with the GHs and CBMs, other CAZymes may also be involved in the deconstruction of cellulose and hemicellulose, most importantly the lytic polysaccharide monooxygenases (LPMO, CAZy family AA10), xylan esterases (CE), and lyases (PL)^4^.

Microorganisms are the main producers of enzymes that decompose cellulose and hemicelluloses in soils, which makes them the most important players in plant biomass decomposition^4^,^10^. Typically, it is assumed that fungi are the major decomposers of complex plant biopolymers in soils^11^. However, these assumptions are mostly based on results obtained with pure cultures and may not reflect reality; recent results indicate that bacteria also significantly contribute to decomposition^3^,^12^. Numerous bacterial strains have been isolated from soils and described as capable of degrading cellulose^10^,^13^,^14^. Most of these strains belong to the phyla *Firmicutes*, *Actinobacteria*, *Proteobacteria* and *Bacteroidetes*^4^,^9^,^10^,^15^,^16^. New molecular methods such as genomics and metagenomics are currently shedding more light on the role that various bacterial taxa play in polysaccharide decomposition^17^. Studies using stable isotope probing in combination with metagenomics have indicated numerous genera from multiple bacterial phyla as real cellulose decomposers^3^,^12^, and the analysis of bacterial genomes demonstrated the presence of genes encoding for potential cellulases in nearly all major bacterial phyla^18^. The presence of genes in microbial genomes, however, only indicates a theoretical capability and does not provide evidence of the actual production of such proteins in the presence of lignocellulose. For example, despite the common occurrence of cellulolytic genes in the genomes of *Actinobacteria*^19^, only a few environmental isolates were able to decompose this biopolymer^13^. Fortunately, gene expression and proteome analyses are technically feasible and can provide such evidence^20^,^21^. In particular, the combination of proteomics and genomics appears to be suitable to describe the genomic potential for lignocellulose decomposition and the expression of related genes.

The aim of this study was to explore the potential of bacteria from forest soil and litter to decompose cellulose and hemicelluloses and to further define their contributions to the decomposition of plant biomass in these ecosystems. Pure culture isolation, cultivation and enzyme assays were used to identify active bacterial taxa and to screen and select representative cellulolytic isolates. Based on the analysis of bacterial abundance in the studied environment^22^, three isolates belonging to the most abundant bacterial taxa, *Mucilaginibacter* L294, *Pedobacter* O48 and *Luteibacter* L214, were selected for detailed analysis of their enzymatic systems combining whole genome sequencing and annotation combined with GeLC-MS/MS to characterize their proteomes during the growth on plant biomass and on microcrystalline cellulose. We hypothesized that bacteria inhabiting forest litter and soil are able to efficiently decompose cellulose, as well as other plant polysaccharides, but are relatively rare. Due to the metabolic costs of enzyme production, complex multi-component enzymatic systems involved in polysaccharide hydrolysis are likely subject to strict substrate-dependent production of their components.

## Results

### Cellulolytic bacteria in forest soil and litter

Cellulolytic activity was exhibited by approximately 12% of bacterial colonies that emerged in plate cultures on minimal medium with carboxymethylcellulose as the carbon source after staining with Congo Red in samples from litter as well as from organic soil (see Supplementary Fig. S1). A total of 115 of such isolates were retained and sequencing identified 42 unique operational taxonomic units (OTUs) at a 99% similarity level. The OTUs belonged to 22 bacterial genera from the phyla *Actinobacteria*, *Bacteroidetes*, *Proteobacteria* and *Firmicutes* (see Supplementary Table S1 and Supplementary Fig. S2). The OTUs to which the identified cellulolytic bacteria belonged exhibited higher relative abundance in litter, where they represented 17.6% of all sequences, compared with 3.7% in the soil (Supplementary Table S1). Although not all the isolates were able to grow under our experimental conditions, several showed enzymatic activities involved in the deconstruction of cellulose and hemicellulose when cellulose was the sole carbon source (see also Supplementary Table S1 and Supplementary Fig. S1). Based on these functional screenings, the following three isolates belonging to those genera highly abundant in the forest topsoil at the study site^22^ were selected for the further analysis: *Mucilaginibacter* L294 and *Pedobacter* O48 belonging to *Bacteroidetes* and the Gammaproteobacterium *Luteibacter* L214. The sequences of the genera *Mucilaginibacter*, *Pedobacter* and *Luteibacter* represented 10.6%, 0.7% and 0.7% in the litter horizon in the area of study^22^.

### Cellulolytic and hemicellulolytic potential of selected bacteria

**Figure 1:**
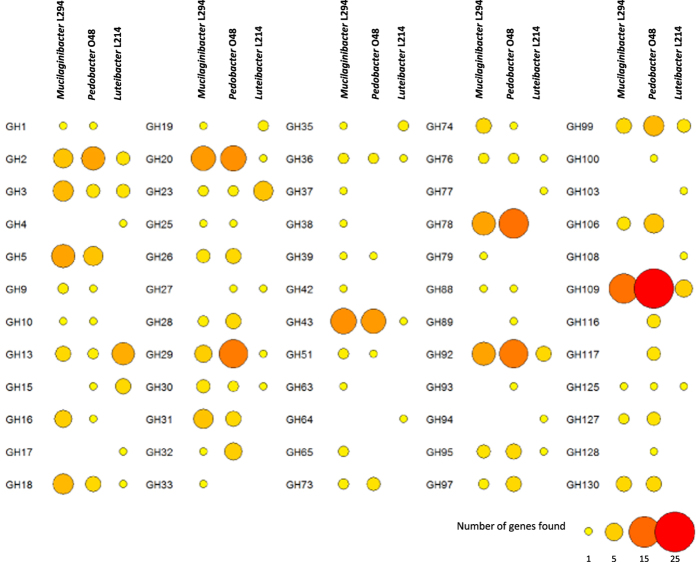
Genes coding for glycosyl hydrolase (GH) families in the genomes of bacteria isolated from forest topsoil. Size and colour of circles indicates predicted gene numbers.

**Figure 2:**
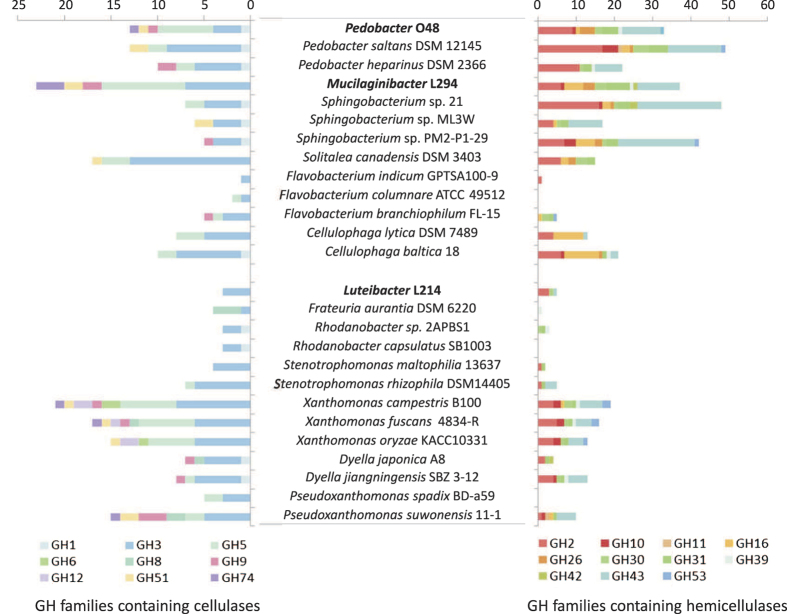
Predicted numbers of glycosyl hydrolases in the genomes of studied isolates from forest topsoil (in bold) and in related bacteria. Only GH families containing enzymes involved in the degradation of cellulose and hemicelluloses are shown. Isolates studied are marked in bold.

The size of the *Mucilaginibacter* L294 genome was estimated at 5.261 Mb with 4698 predicted coding sequences, in *Pedobacter* O48 6.380 Mb with 5399 predicted coding sequences and in *Luteibacter* L214 4.989 Mb with 4415 predicted coding sequences (Supplementary Table S2). Both *Pedobacter* O48 and *Mucilaginibacter* L294 showed high numbers of predicted glycosyl hydrolases (GHs) in their genomes (191 or 3.5% and 161 or 3.4%, respectively), while only 57 were predicted in *Luteibacter* L214 (1.3% of genes). In total, putative genes belonging to 60 GH families were identified in the genomes (Fig. 1; Supplementary Table S3) of which 16 families were present in all three genomes, 5 families were found only in *Mucilaginibacter* L294, 6 only in *Pedobacter* O48 and 7 only in *Luteibacter* L214. The numbers of putative genes identified for each family were variable and isolate-specific. The families including genes encoding for potential cellulases (GH1, GH5, GH9, GH51 and GH74) were only observed in *Pedobacter* O48 and *Mucilaginibacter* L294, and only the putative cellulases belonging to GH3 were also found in *Luteibacter* L214. Genes involved in hemicellulose deconstruction and belonging to families GH2, GH30 and GH43 were abundant in all genomes whereas other hemicellulolytic genes from families GH10, GH16, GH26, GH31 and GH39 were only found in the *Bacteroidetes*. Similar results were found when the gene complement of the isolates was compared with related genome-sequenced bacteria. Those related to *Luteibacter* L214 also showed low numbers of potential cellulases and hemicellulases (Fig. 2). Genes encoding for CBM domains involved in cellulose and hemicellulose binding (CBM9 and CBM44) were identified in all genomes. Moreover, seven genes encoding for domains belonging to families CBM6 and CBM37 were identified in the *Pedobacter* O48 genome, two genes involved in cellulose binding (CBM30) were annotated in the *Mucilaginibacter* L294 and two genes involved in xylan binding (families CBM22 and CBM35) were observed only in *Luteibacter* L214. Multiple carbohydrate esterases (CE) were also detected in all the bacterial genomes, including families encoding for enzymes that act on xylan (CE1, CE3, CE4, CE10 and CE14). No genes encoding for oxidative cellulases (AA10) were identified (Supplementary Tables S2 and S3).

Most of the genes encoding for putative cellulases and hemicellulases were randomly distributed along the genomes of the three bacteria. In general, genes encoding for CAZymes were flanked by numerous putative proteins involved in signal transduction, nutrient binding and transport. Most of these genes were annotated as TonB-dependent receptors and ABC transporters as well as ATP-binding proteins and permeases. In some cases, such as in *Mucilaginibacter* L294, a putative GH9 was adjacent to seven such genes, and two GH5 genes were associated with three of these putative transporters. TonB-dependent receptors have been previously identified as plant carbohydrate scavengers in some bacteria from *Bacteroidetes* and *Proteobacteria* phyla^23^. The proximity of these putative transporter with genes encoding for CAZymes have been suggested in other bacterial species as an indicator of their involvement in the transport of sugars released by the enzymes^24^. Moreover, numerous transcriptional regulators from diverse families (mainly AraC, LacI, ArsR, TetR, and GntR) and sigma and anti-sigma factors were found surrounding CAZyme genes, which has also been reported in some cellulolytic strains in *Bacteroidetes* and *Firmicutes*^25^.

Interestingly, putative genes encoding for Type IV pili involved in cellulose binding were found in the genomes of *Luteibacter* L214 (33 genes) and *Pedobacter* O48 (5 genes), but not in *Mucilaginibacter* L294. These proteins have been implicated in adherence to cellulose in *Fibrobacter* and *Ruminococcus* species^25^,^26^ and play an important role in competition with other cellulolytic species for adhesion to cellulose.

### General protein expression profiling during growth on cellulose and plant biomass

**Figure 3:**
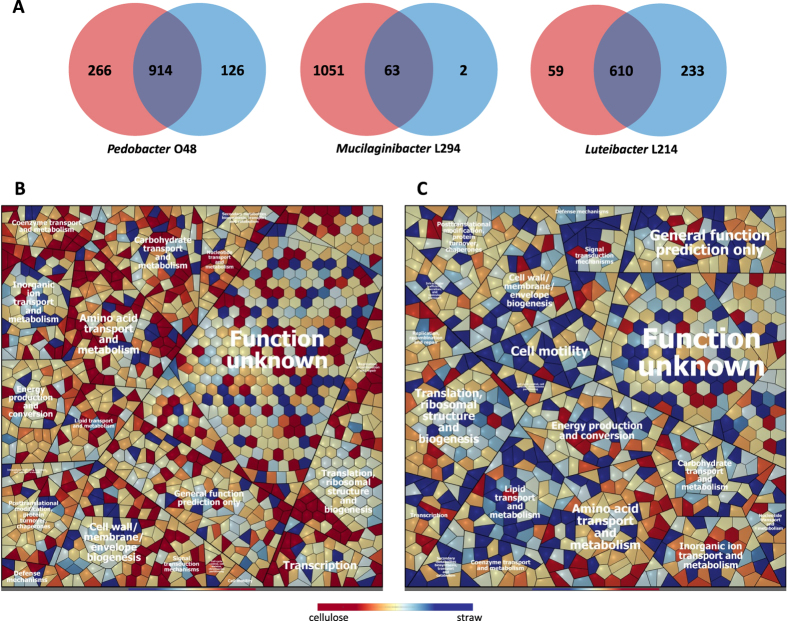
Identification of proteins in the secretomes of the analyzed bacteria. (**A**) Venn diagrams depicting identified proteins during growth on cellulose (red) and wheat straw (blue). Voronoi treemap visualization of protein expression patterns of *Pedobacter* O48 (**B**) and *Luteibacter* L214 (**C**) during growth on cellulose (red) and wheat straw (blue). Functional classification of proteins was carried out by *Prophane* 2.0 and RAST. Each cell represents a protein, and proteins are clustered according to their function. Functional classes are separated by thicker black lines. Due the limited growth of *Mucilaginibacter* L294 on straw, comparison of expression profiles for this bacterium was impossible.

Analysis by GeLC-MS/MS allowed us to identify 1306 proteins in the *Pedobacter* O48 proteome, 1116 in the *Mucilaginibacter* L294 proteome, and 902 in the *Luteibacter* L214 proteome (Fig. 3A; see also the Supplementary Files SF1, SF2, SF3). Approximately 70% of proteins were expressed in both cellulose and wheat straw-supplemented cultures of *Pedobacter* O48 and *Luteibacter* L214, respectively. In contrast, the overlap between identified proteins of *Mucilaginibacter* L294 grown on cellulose and those grown on straw was only 6%. This was due to limited growth on straw (data not shown), and thus a comparison of expression profiles was impossible. In *Pedobacter* O48, 266 proteins were found exclusively in the cellulose cultures, and 126 were found only in wheat straw cultures (Fig. 3A; Supplementary File SF1). The majority of 914 proteins expressed in both cultures showed higher relative abundance in cellulose cultures (Fig. 3B). In contrast to *Pedobacter* O48, *Luteibacter* L214 cultures showed fewer proteins specific for growth on cellulose – 59 – whereas 233 were exclusively observed on straw (Fig. 3A; Supplementary File SF3). Most of the proteins expressed in both cultures showed higher relative abundance on straw (Fig. 3C). Proteins involved in membrane transport, chemotaxis, and mobility and those with different subroles within carbohydrate metabolism were more frequently expressed in straw, demonstrating the substantial influence of complex carbon sources on metabolism.

### Carbon source-dependent expression of proteins involved in cellulose and hemicellulose degradation

An overview of identified CAZymes found in the genomes and proteomes of the three analyzed bacteria is summarized in Supplementary Table S3 online. The percentages of CAZymes found in cultures grown on wheat straw were slightly higher than in cultures grown on crystalline cellulose: 21.4% vs. 20.0% in *Pedobacter* O48 and 16.6% vs. 15.4% in *Luteibacter* L214. While none of the very few identified proteins of *Mucilaginibacter* L294 cultivated in the presence of straw were annotated as CAZymes, 24% of predicted CAZymes were observed on cellulose.

**Figure 4:**
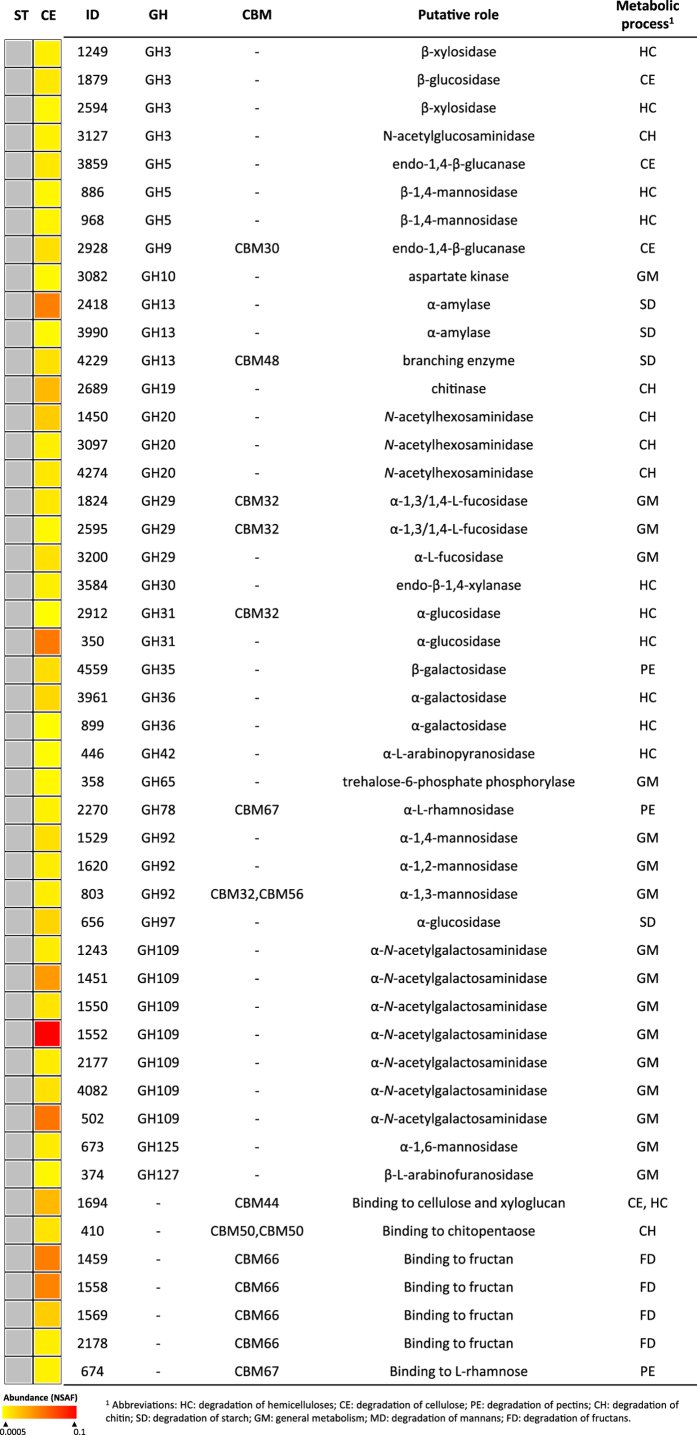
Glycosyl hydrolases and carbohydrate binding modules in the proteomes of *Mucilaginibacter* L294 growing in wheat straw (ST) and cellulose (CE) as carbon sources. Protein abundance is color-coded increasing from yellow to red. Grey cells indicate that no CAZymes were found on ST in this bacterium.

In *Mucilaginibacter* L294, GHs belonging to 44% of predicted families were recorded on cellulose (Fig. 4; Supplementary Table S3). Among these, eight proteins were from three GH families containing cellulolytic enzymes (GH3, GH5 and GH9). Two of them (ID3859 and ID2928) showed similarity to two characterized endoglucanases (GH5 and GH9, respectively), and the latter also contained the cellulose-binding CBM30 domain. Another cellulose-binding domain (CBM44) was also abundant. Five proteins from four different GH families (GH10, GH30, GH31 and GH42) involved in hemicellulose decomposition were also identified during growth on cellulose, including a putative endo-1,4-β-xylanase from GH30 (Fig. 4). In addition, several glycosyl hydrolases from families GH109, GH13, GH19 and CBM66 were identified in cellulose-grown *Mucilaginibacter*, although these families contain neither cellulases nor hemicellulases.

**Figure 5:**
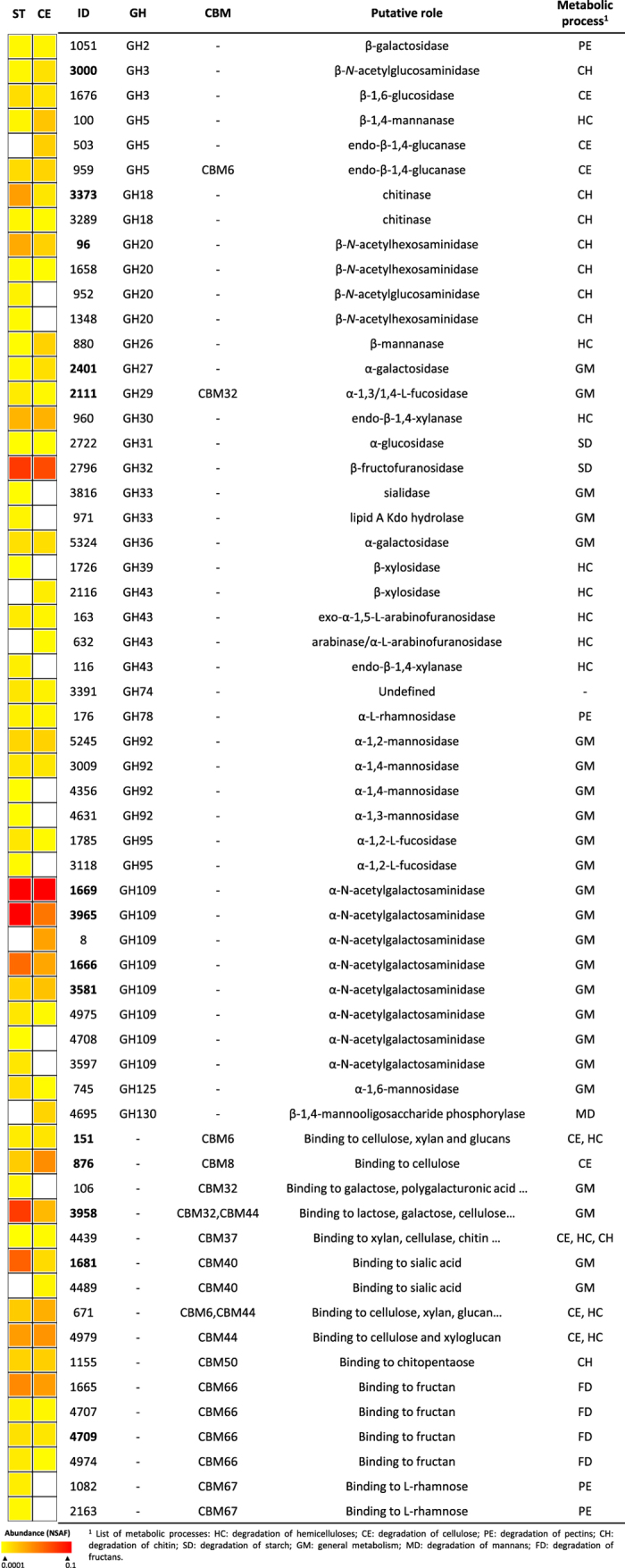
Glycosyl hydrolases and carbohydrate binding modules in the proteomes of *Pedobacter* O48 growing in wheat straw (ST) and cellulose (CE) as carbon sources. Protein abundance is color-coded increasing from yellow to red, and CAZymes not recorded are in white. Proteins found in both carbon sources and showing significantly differences (p < 0.01) in expression are marked with ID number in bold.

Fourteen proteins from nine GH families involved in cellulose and hemicellulose decomposition (six and eight proteins, respectively) were found in the proteome of *Pedobacter* O48 (Fig. 5; Supplementary File SF1). Two endo-1,4-β-glucanases from the family GH5 were detected: ID959 that contained a domain for cellulose binding (CBM6) and ID503, which was only produced on cellulose. Two hemicellulases encoding for two putative endo-1,4-β-xylanases (GH 30 and GH43) were also found, but the latter was identified only on straw. Seven proteins containing domains involved in cellulose and/or xylan binding (CBM6, CBM8, CBM37 and CBM44) were detected in both cultures. In general, the proteome recovered from these straw cultures was richer in GHs, expressing twelve specific GHs compared to five GHs on cellulose. Straw cultures also expressed more proteins containing GHs and CBMs for the cleavage and binding of fucose, galactose, rhamnose and mannose residues present in the hemicelluloses and pectins. Despite the absence of the corresponding polysaccharides, around the 60% of these GHs and CBMs identified in the proteome were also expressed on cellulose (Fig. 5).

**Figure 6:**
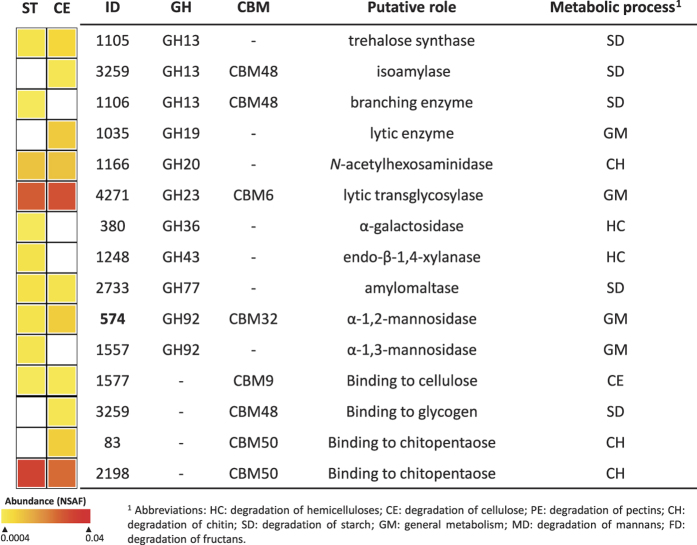
Glycosyl hydrolases and carbohydrate binding modules in the proteomes of *Luteibacter* L214 growing in wheat straw (ST) and cellulose (CE) as carbon sources. Protein abundance is color-coded increasing from yellow to red, and CAZymes not recorded are in white. Proteins found in both carbon sources and showing significantly differences (p < 0.01) in expression are marked with ID number in bold.

Despite the ability of *Luteibacter* L214 to efficiently degrade CMC, no proteins from GH families with cellulolytic activity were detected in its proteome (Fig. 6; Supplementary File SF3). Only one hemicellulase, identified as endo-1,4-β-xylanase (GH43), was expressed on straw. On the other hand, two proteins showing domains for cellulose binding (CBM6 and CBM9) were expressed in both cultures. One of these, ID4271, showed also the catalytic domain of a GH23 family type. Six of fifteen proteins encoding for GHs and CBMs were common for both carbon sources and four proteins encoding for families not involved in cellulose decomposition (GH13, GH19, CBM48 and CBM50) were expressed only during growth on cellulose.

### Occurrence of other glycoside hydrolases and other CAZymes

Other GHs involved in general bacterial metabolism such as glycan biosynthesis and its catabolism, e.g., mannosidases (GH92) and *N*-acetylgalactosaminidases (GH109), were found in all bacterial cultures. Some of these proteins were specific for only one of the substrates, while others were abundant in both (Figs 5 and 6). A similar number of auxiliary activity enzymes (AAs) was found on both carbon sources for *Pedobacter* O48 and *Luteibacter* L214; higher numbers of carboxyl esterases were found on straw (Supplementary Table S3 and Supplementary Files SF1 and SF3). Single polysaccharide lyase (PL22) was expressed by *Luteibacter* L214 whereas PLs were much more common in the *Mucilaginibacter* L294 genome. The number of glycosyl transferases (GTs) found in all proteomes was low, comprising less than 10% of GTs predicted by genomic analysis.

### Expression of other proteins involved in cellulose utilization

In general, numerous proteins identified as transcriptional regulators were abundantly identified during growth on cellulose in *Pedobacter* O48 and *Mucilaginibacter* L294; meanwhile, transcriptional regulators were mostly abundant or only identified during growth in straw in the case of *Luteibacter* L214. Proteins annotated as TonB-like receptors were found during growth on both carbon sources, and they were more numerous and abundant on straw than on cellulose in *Pedobacter* O48 and *Luteibacter* L214. ABC transporters were detected in all proteomes, but they were more abundant in *Luteibacter* L214 cultures on wheat straw than on cellulose. Interestingly, a large number of proteins associated with the formation of Type IV pili were identified in *Luteibacter* L214, most of which were more abundant on straw than on cellulose. The major pilin PilA (ID1452) was one of the most abundant proteins in straw-cultured *Luteibacter* L214. Additionally, other proteins involved in chemotaxis and motility were upregulated in the presence of straw as well (Fig. 3C; Supplementary File SF3).

## Discussion

Although numerous strains from diverse soils have been previously isolated and identified as cellulolytic, these surveys are severely biased towards a limited set of taxa from specific ecosystems such as compost, cow rumen or bioreactor enrichment cultures^9^,^10^. In this study, members of 22 bacterial genera isolated from forest soil and litter were demonstrated to decompose cellulose. Our results confirm our first hypothesis, showing that cellulolytic abilities are common in bacteria from forest topsoil and especially litter. Despite limitations in isolation techniques for some bacterial groups with low culturability (e.g., the *Acidobacteria* and other slow-growing taxa), our isolation efforts yielded members of several genera highly abundant in the studied ecosystem, such as *Mucilaginibacter*, *Pedobacter*, *Streptomyces*, *Sphingomonas*, *Burkholderia* or *Luteibacter*^22^. Although cellulolytic members were previously recorded in some of these genera^3^,^12^,^27^, these records were often indirect (such as by the means of stable isotope probing), and for others, such as *Humibacter*, *Plantibacter*, *Frondihabitans* and *Luteibacter*, cellulolytic members have not yet been described. Several isolates (approximately 21% of the selected strains) showed an ability to grow on crystalline cellulose as the sole C source and to produce enzymes involved in cellulose and hemicellulose degradation. In contrast to the second part of our hypothesis, the strains showing cellulolytic properties in the studied environment were rather abundant, representing as much as 17% of the total community in litter. These findings support the role of bacteria as important players in the decomposition of plant biomass in forest topsoil, as proposed based on stable isotope probing^12^. It also shows that the spatial separation of bacterial taxa in forest topsoils^22^,^28^ has a functional aspect and reflects cellulose availability in litter and soil.

The recent analysis of bacterial genomes showed that genes encoding for GH families containing cellulases are present in 24% of the sequenced prokaryotes^18^. The true cellulolytic bacteria typically contain several CAZyme-encoding genes from families GH1 and GH3 encoding for β-glucosidases together with genes encoding for endo- and exocellulases from other GH families^4^. This is also the case of *Mucilaginibacter* L294 with 8 genes from GH1 and GH3, and 17 genes from families GH5, GH9, GH51 and GH74 in its genome and *Pedobacter* O48 with 4 genes from GH1 and GH3 and 9 from the families containing the “true” cellulases. These gene suites make them typical cellulose decomposers. The correlation between a high number of CAZymes in the genome and the ability for plant biomass degradation found in some bacteria has been predicted by several authors^24^,^29^. On the other hand, *Luteibacter* L214, with only three β-glucosidase genes from family GH3, would be classified as a noncellulolytic opportunistic bacterium according to the above rules.

Genomics can provide information on the functional potential of microorganisms, but it does not imply that the predicted phenotype is expressed^30^,^31^. The present study demonstrated clearly that the full complement of cellulases and hemicellulases was neither expressed on crystalline cellulose nor on complex lignocellulose represented by wheat straw. On the other hand, the comparison with characterized CAZymes still revealed a high level of functional redundancy among the GHs with multiple predicted endoglucanases, β-glucosidases, endoxylanases, β-xylosidases and mannosidases (Figs 4, 5, 6; Supplementary Table S3). This redundancy may indicate synergistic effects of enzymes on decomposition as was previously observed by other authors^15^. Our findings also confirm our hypothesis, showing that cellulolytic bacteria are in no way specialist cellulose decomposers and have the potential to utilize wider spectra of polysaccharides as the complexity of their enzymatic systems is comparable to that found in fungi^31^.

Interestingly, traditional cellulolytic proteins were not found in the proteomes of *Luteibacter* L214 despite the fact that the strain was able to grow on microcrystalline cellulose and also showed cellulolytic activity when grown on filter paper. The fact that the GH complement of *Luteibacter* L214 was similar to the related *Frauteria* and *Rhodanobacter* (Fig. 2) suggests that this is typical for a larger group of *Gammaproteobacteria*. Apparently, its cellulolytic phenotype is a result of the activity of proteins not previously predicted to be involved in this process. In recent years, multiple novel proteins involved in cellulose cleavage have been described in both fungi and bacteria^6^,^32^. One of these is a new endo-acting cellulase whose active site displayed structural properties similar to the proteins of the GH23 family and has been designated as a lytic transglycosylase^33^. In our study, the GH23 protein was highly expressed in *Luteibacter* L214 cultures (Fig. 6) and showed remarkable sequence similarity to lytic transglycosylases. This protein contained the domain CBM6 with a potential to bind cellulose, xylan and glucans, further supporting its role in the hydrolysis of cellulose and possibly other polysaccharides. The importance of cellulose decomposition for this bacterium is also supported by the presence of the type IV pili that mediate adhesion to cellulose^25^,^34^. However, further work would be needed to ultimately confirm the involvement of the GH23 family protein in the decomposition of polysaccharides.

Despite slight differences in GH and CBM expression in the two studied substrates, most of them were expressed in both cultures. The expression of proteins decomposing various polysaccharides in cellulose cultures may seem strange when considering the metabolic costs of production of these “unnecessary” enzymes. However, if we consider that cellulose typically occurs in nature along with other plant cell wall polysaccharides, cellulose or its degradation products may indicate the presence of complex lignocellulose and act as a signal for their production. Such general induction may be more efficient than a complex regulatory system recognizing various polysaccharides if we consider the variability of these structures. So far, the regulatory systems that respond to the presence of cellulose degradation products such as cellobiose and other cellodextrins remain unknown in most bacteria^35^. However, some studies showed that certain bacteria express glycosyl hydrolases even during growth on glucose^35^,^36^. One of these studies showed that glucose induced similar CAZymes as cellulose in *Clostridium cellulolyticum* and that cellulose was more quickly degraded when glucose was present, hypothesizing that this basal expression may mediate a faster response of the bacteria to cellulose availability^36^. The presence of numerous and diverse transcriptional regulators, sigma and anti-sigma factors, TonB-like receptors and transport-related proteins flanking the CAZymes suggest that lignocellulose degradation is indeed a process with complex regulation, as has already been demonstrated for the bacterial genera *Caldicellulosiruptor*, *Fibrobacter* or *Ruminicoccus*^24^,^25^,^26^. In the same way, the presence of other proteins involved in chitin degradation (chitinases and *N*-acetylglucosaminidases) and starch catabolism (such as amylases, maltases and fructofuranosidases) in the bacterial proteomes during growth in cellulose that we have observed, suggests that cellulases and hemicellulases may share their regulatory systems with other proteins, as proposed by some authors^37^.

Our study shows that cellulose hydrolysis is a relatively common trait of bacteria inhabiting forest litter and soil. Two of the most abundant cellulolytic bacteria, *Mucilaginibacter* and *Pedobacter*, are apparently active players in plant biomass decomposition, as revealed by the rich suite of proteins active in cellulose and hemicellulose decomposition. Interestingly, the cellulolytic phenotype is not limited to those bacterial taxa that possess and express traditional cellulases as seen in the case of *Luteibacter* L214 expressing cellulose-binding transglycosylase. Furthermore, the fact that multiple proteins exclusively expressed by bacteria on cellulose have unknown functions indicates the need for further studies.

## Methods

### Isolation, identification and selection of bacterial strains

Bacteria were isolated from the forest floor of a temperate oak forest (*Quercus petraea*) in the Czech Republic. Litter chemistry and decomposition processes as well as the composition of bacterial communities in the litter and soil were previously studied in the selected area^2^,^22^,^38^. Samples were collected in four defined plots (10 m^2^, approximately 100 m apart from each other) at the sampling site. Four samples were manually collected at each sampling plot from both litter and organic soil horizons. Litter samples were cut into small pieces and soil organic horizon material was sieved using a 2-mm sieve. Samples (0.5 g) of litter and soil were extracted with Ringer solution (100 mL g^−1^) for 15 min on a shaker in duplicate. One hundred fifty microliters of each suspension were plated on agar CMC medium (2 g L^−1^ yeast extract, 5 g L^−1^ carboxymethylcellulose (CMC), 50 mg L^−1^ of cycloheximide, pH 7.0) and incubated at 25 °C. After 7 days, pure bacterial strains (approx. 500 from soil and 500 from litter) were isolated from emerging colonies using the same media. Agar plates were stained with 0.1% Congo red^39^, and only bacterial colonies showing clear halos indicating carboxymethylcellulose degradation (115 isolates) were selected.

Isolates were identified by 16S rRNA gene sequencing using the primers 530F and 1100R^40^. To avoid redundant analysis of isolates of the same or closely related strains, sequences were clustered into OTU at a 99% similarity level using Usearch^41^ in the SEED pipeline^42^. Representative isolates from each OTU was selected for further analysis and 16S rRNA genes were sequenced with the primers 27F and 1492R^40^. Long sequences from selected OTUs were used for isolate identification using the EzTaxon server^43^ (http://www.ezbiocloud.net/eztaxon) and deposited in Genbank database under the accession numbers KR181794-KR181835.

The identified isolates were tested for hydrolytic enzyme activity and the ability to decompose cellulose and hemicellulose. Isolates were pre-grown in liquid CMC medium for 24 h and inoculated onto liquid minimal medium (MM, pH 7.0; 2 g L^−1^ NH_4_H_2_PO_4_, 0.6 g L^−1^ KH_2_PO_4_, 0.5 g L^−1^ MgSO_4_·7H_2_O, 0.4 g L^−1^ K_2_HPO_4_, and 10 mL L^−1^ of trace elements solution). The trace element solution contained 7.40 g L^−1^ CaCl_2_·2H_2_O, 1.20 g L^−1^FeSO_4_·7H_2_O, 0.66 g L^−1^ZnSO_4_·7H_2_O, 0.5 g L^−1^MnSO_4_·4H_2_O, 0.10 g L^−1^CoCl_2_·6H_2_O, and 1 mg L^−1^ thiamine. Cellulose filter paper strips were added to the medium as the sole carbon source, and cultures were grown for 7 days at 25 °C on an orbital shaker. Enzyme assays were performed as described previously^44^ for the following enzymes: cellobiohydrolase (exocellulase), β-glucosidase, β-xylosidase, α-arabinosidase, glucuronidase, β-galactosidase, α-glucosidase and β-mannosidase. Briefly, the activities were measured using methylumbelliferol(MUF)-based substrates on a microplate reader (Infinite, TECAN, Austria), with an excitation wavelength of 355 nm and an emission wavelength of 460 nm. Calibration of product development was based on standard curves with a range of MUF concentrations added to the sample. Strains belonging to different bacterial genera that exhibited high enzyme activities in this screening (*Mucilaginibacter* L294, *Pedobacter* O48, and *Luteibacter* L214) as well as high abundance in the studied ecosystem^22^ were selected for genome sequencing and proteome analyses.

### DNA extraction, whole-genome sequencing and genome analysis

Total genomic DNA was extracted from three selected strains grown in GYM media (4 g L^−1^ glucose; 4 g L^−1^ yeast extract; 10 g L^−1^ malt extract; pH 7.0) with the UltraClean Microbial DNA Isolation Kit (MoBio Laboratories, Carlsbad, CA, USA), and sequencing was performed on the Illumina MiSeq platform in a paired-end 2 × 250 bp run. The sequence data were assembled using SPAdes 3.0^45^, and draft genomes were obtained. Draft genome sequences were deposited in Genbank under the accession numbers LGEK00000000, LGEL00000000 and LGRQ00000000. Gene annotation was performed using Rapid Annotations Subsystems Technology (RAST) 4.0^46^,^47^. To identify the CAZymes, translated proteins from the predicted open reading frames were analyzed with dbCAN^48^. Information about the carbohydrate-active enzyme content in the genomes of closely related bacteria was obtained from the CAZy database^7^.

### Protein expression and 1D gel-LCMS/MS analysis of proteins

To analyze protein expression, bacteria were pre-grown in GYM media for 24 h, and 1 mL of culture was inoculated in triplicate on 1 L of MM containing 0.5% w/v of either microcrystalline cellulose (Serva, Heidelberg, Germany) or wheat straw that was finely milled and repeatedly washed with hot water to remove low-molecular-mass compounds while retaining plant cell wall biopolymers. Cultures were incubated for 7 days at 25 °C in an orbital shaker. After harvest, cultures were centrifuged and proteins in supernatant were precipitated with 10% w/v trichloroacetic acid and resuspended in 8 M urea / 2 M thiourea buffer. Protein concentration was measured in every sample with Roti-Nanoquant (Carl Roth, GmbH, Germany), and 25 μg were separated by 1D-SDS-PAGE using Criterion TGX Precast Gels (BioRad Laboratories, Hercules, CA, USA) (see Supplementary Fig. S3). Lanes were cut in ten equidistant pieces and subjected to trypsin digestion as previously described^49^. Peptide mixtures were separated by RP chromatography using a nanoAQUITY UPLC system (Waters, Milford, MA, USA). Peptides were loaded onto a trapcolumn and separated on the analytical column using a binary 80 min gradient of buffer B (99.9% ACN, 0.1% acetic acid) at a constant flow rate of 400 nL min^−1^. The UPLC system was coupled to an LTQ Orbitrap mass spectrometer (Thermo Fisher Scientific, Waltham, MA, USA). Full survey scans were recorded in the Orbitrap (m/z range from 300 to 2,000) with a resolution of 30,000 and lock mass option enabled. MS/MS experiments in the LTQ XL were performed for the five most abundant precursor ions (CID), excluding unassigned charge states and singly charged ions. Dynamic exclusion was enabled after 30 s. For protein identification, spectra were searched against databases of *Pedobacter* O48, *Mucilaginibacter* L294 and *Luteibacter* L214, respectively, containing sequences of all predicted proteins from their genomes. Database searches were performed using Sorcerer SEQUEST (Version v. 27 rev. 11, Thermo Scientific) and Scaffold 4.0.5 (Proteome Software, Portland, OR, USA). Protein quantification was based on the normalized spectrum abundance factor (NSAF), which is calculated as the number of spectral counts (SpC) that identify a protein, divided by protein length (L), and divided by the sum of SpC/L for all proteins in the experiment^50^. Statistical analysis was performed using MeV v4.8.1^51^. Functional prediction and classification of proteins was completed using the in-house developed analysis pipeline Prophane 2.0 (http://www.prophane.de)^52^ and the RAST annotation server. Voronoi treemaps were generated using Paver (Decodon, Greifswald, Germany; http://www.decodon.com/). Sequences of the identified proteins and those previously predicted to be GHs with dbCAN were compared with the characterized sequences deposited in the CAZy database for the GH families found in the proteome for function prediction. For this purpose, sequences were aligned with MUSCLE 3.7^53^, and trees based on the maximum likelihood were constructed with PhyML 3.0^54^. Where possible, the putative role for the identified GHs was directly assigned based on the closest sequences in the family tree whose functional roles were known. The mass spectrometry proteomics data have been deposited to the ProteomeXchange Consortium via the PRIDE^55^ partner repository with the dataset identifier PXD003844.

## Additional Information

**How to cite this article**: López-Mondéjar, R. *et al*. Cellulose and hemicellulose decomposition by forest soil bacteria proceeds by the action of structurally variable enzymatic systems. *Sci. Rep*. **6**, 25279; doi: 10.1038/srep25279 (2016).

## Supplementary Material

Supplementary Information

Supplementary Table 1

Supplementary Table 2

Supplementary Table 3
