# Supplementary material for: Cellulose and hemicellulose decomposition by forest soil bacteria proceeds by the action of structurally variable enzymatic systems
Source: Sci Rep. 2016 Apr 29;6:25279. doi: 10.1038/srep25279 (PMC4850484; doi:10.1038/srep25279)

Supplementary Information

Cellulose and hemicellulose decomposition by forest soil bacteria proceeds by the action of structurally variable enzymatic systems

Rubén López-Mondéjar[1]*, Daniela Zühlke[2], Dörte Becher[2], Katharina Riedel[2], and Petr Baldrian[1]

[1]Laboratory of Environmental Microbiology, Institute of Microbiology of the ASCR, Prague, 14220, Czech Republic

[2]Institute of Microbiology, Ernst-Moritz-Arndt-University of Greifswald, Greifswald, D-17487, Germany

*Address correspondence to Rubén López-Mondéjar, rubenlopezmondejar@gmail.com, mondejar@biomed.cas.cz

Supplementary Table S1. List of the different bacterial strains isolated and identified via 16S from the forest soil and their production of extracellular enzymes. Isolates were also assigned to bacterial OTUs found in the same ecosystem based on a previous study (1). Abundance data of OTUs containing the isolate are shown for litter and soil. Abbreviations of enzymes: bG – β-glucosidase; aG – α-glucosidase; CBH – cellobiohydrolase; bX – β-xylosidase; aAr – α-arabinosidase; bM – β-mannosidase; Glu – glucuronidase; bGal – β-galactosidase.

**Isolate**[1]	**Genus**	**Accession no.**	**Closest type strain** [2]	**Pairwise Similarity (%)**	**OTU**[3]	**Abundance in litter (%)**[4]	**Abundance in organic soil (%)**	**bG**[5]	**aG**	**CBH**	**bX**	**aAr**	**bM**	**Glu**	**bGal**
Actinobacteria															
L395	*Plantibacter*	KR181794	*P. auratus* IAM 18417	98.1	B04270	0.00	0.00	ng	ng	ng	ng	ng	ng	ng	ng
O406	*Humibacter*	KR181795	*H. antri* D7-27	98.5	B00615	0.03	0.01	ng	ng	ng	ng	ng	ng	ng	ng
O266	*Arthrobacter*	KR181796	*A. livingstonensis* L12	97.2	B00595	0.02	0.00	ng	ng	ng	ng	ng	ng	ng	ng
L242	*Curtobacterium*	KR181797	*C. flaccumfaciens* LMG 3645	99.9	B00314	-	-	ng	ng	ng	ng	ng	ng	ng	ng
L370	*Frondihabitans*	KR181798	*F. peucedani* RS-15	98.7	B00262	0.72	0.12	ng	ng	ng	ng	ng	ng	ng	ng
O52	*Streptacidiphilus*	KR181799	*S. carbonis* DSM 41754	98.8	B00245	0.26	0.09	ng	ng	ng	ng	ng	ng	ng	ng
O258	*Streptomyces*	KR181800	*S. griseorubiginosus* NBRC 13047	99.6	B00245	0.26	0.09	nt	nt	nt	nt	nt	nt	nt	nt
O65	*Streptomyces*	KR181801	*S. laculatispora* BK166	99.9	B00245	0.26	0.09	nt	nt	nt	nt	nt	nt	nt	nt
L321	*Streptomyces*	KR181802	*S. abietis* A191	98.6	B00245	0.26	0.09	nt	nt	nt	nt	nt	nt	nt	nt
L124	*Pilimelia*	KR181803	*P. columellifera* MB-SK 8	99.4	B00245	0.26	0.09	nt	nt	nt	nt	nt	nt	nt	nt
Bacteroidetes															
L294	*Mucilaginibacter*	KR181804	*M. rigui* WPCB133	99.4	B01451	0.06	0.03	+	+	+	+	+	+	-	+
L356	*Mucilaginibacter*	KR181805	*M. soyangensis* HME6664	98.2	B00015	4.48	0.89	ng	ng	ng	ng	ng	ng	ng	ng
O48	*Pedobacter*	KR181806	*P. panaciterrae* Gsoil 042	98.3	B00056	0.69	0.05	+	+	+	+	+	+	-	+
O230	*Chitinophaga*	KR181807	*C. arvensicola* DSM 3695T	99.1	B00951	0.01	0.01	ng	ng	ng	ng	ng	ng	ng	ng
Alphaproteobacteria															
L205	*Sphingomonas*	KR181808	*S. asaccharolytica* IFO 15499-T	99.9	B00237	0.05	0.02	+	-	+	+	+	+	-	+
L367	*Sphingomonas*	KR181809	*S. polyaromaticivorans* B2-7	97.8	B00252	0.24	0.02	ng	ng	ng	ng	ng	ng	ng	ng
Betaproteobacteria															
O49	*Burkholderia*	KR181810	*B. sediminicola* HU2-65W	99.5	B00010	1.84	0.44	nt	nt	nt	nt	nt	nt	nt	nt
O395	*Burkholderia*	KR181811	*B. phenazinium* LMG 2247	99.3	B00010	1.84	0.44	-	+	-	+	-	-	+	-
L578	*Burkholderia*	KR181812	*B. fungorum* NBRC 102489	99.2	B00010	1.84	0.44	-	-	-	-	-	-	+	-
O295	*Burkholderia*	KR181813	*B. udeis* Hg2	99.1	B00435	0.59	0.13	nt	nt	nt	nt	nt	nt	nt	nt
L402	*Burkholderia*	KR181814	*B. sordidicola* S5-B	100.0	B00435	0.59	0.13	ng	ng	ng	ng	ng	ng	ng	ng
L511	*Burkholderia*	KR181815	*B. phytofirmans* PsJN	99.0	B00010	1.84	0.44	-	-	+	+	-	-	-	-
L210	*Variovorax*	KR181816	*V. boronicumulans* BAM-48	99.4	B00332	0.18	0.04	ng	ng	ng	ng	ng	ng	ng	ng
L398	*Variovorax*	KR181817	*V. ginsengisoli* Gsoil 3165	99.4	B00332	0.18	0.04	ng	ng	ng	ng	ng	ng	ng	ng
Gammaproteobacteria															

L241	*Erwinia*	KR181818	*E. billingiae* Eb661	99.5	B00246	-	-	nt	nt	nt	nt	nt	nt	nt	nt
L261	*Stenotrophomonas*	KR181819	*S. rhizophila* DSM 14405	100.0	B02209	0.00	0.02	ng	ng	ng	ng	ng	ng	ng	ng
L214	*Luteibacter*	KR181820	*L. rhizovicinus* LJ96	99.6	B00149	0.73	0.01	+	+	+	+	+	-	-	-
O200	*Dyella*	KR181821	*D. soli* JS12-10	98.7	B00149	0.73	0.01	ng	ng	ng	ng	ng	ng	ng	ng
Firmicutes															
O5	*Bacillus*	KR181822	*B. weihenstephanensis* NBRC 101238	100.0	B00935	0.04	0.06	nt	nt	nt	nt	nt	nt	nt	nt
O35	*Bacillus*	KR181823	*B. anthracis* ATCC 14578	99.7	B00935	0.04	0.06	nt	nt	nt	nt	nt	nt	nt	nt
O339	*Sporosarcina*	KR181824	*S. globispora* DSM 4	99.1	B00089	0.06	0.08	ng	ng	ng	ng	ng	ng	ng	ng
O142	*Cohnella*	KR181825	*C. luojiensis* HY-22R	98.1	B00751	0.00	0.01	ng	ng	ng	ng	ng	ng	ng	ng
O45	*Paenibacillus*	KR181826	*P. aestuarii* CJ25	94.4	B03837	0.00	0.00	nt	nt	nt	nt	nt	nt	nt	nt
O279	*Paenibacillus*	KR181827	*P. pectinilyticus* RCB-08	99.6	B00539	0.02	0.03	nt	nt	nt	nt	nt	nt	nt	nt
O168	*Paenibacillus*	KR181828	*P. vulneris* CCUG 53270T	97.1	B01054	0.01	0.00	nt	nt	nt	nt	nt	nt	nt	nt
O326	*Paenibacillus*	KR181829	*P. rigui* WPCB173	96.5	B01054	-	-	nt	nt	nt	nt	nt	nt	nt	nt
O280	*Paenibacillus*	KR181830	*P. mendelii* C/2	94.9	B00343	0.02	0.01	ng	ng	ng	ng	ng	ng	ng	ng
L304	*Paenibacillus*	KR181831	*P. castaneae* Ch-32	99.2	B00343	0.02	0.01	nt	nt	nt	nt	nt	nt	nt	nt
O283	*Paenibacillus*	KR181832	*P. odorifer* TOD45	96.1	B03156	0.00	0.00	-	-	+	+	+	+	+	+
O399	*Paenibacillus*	KR181833	*P. pini* S22	99.6	B00402	0.01	0.02	nt	nt	nt	nt	nt	nt	nt	nt
O199	*Paenibacillus*	KR181834	*P. tundrae* A10b	99.9	B00402	0.01	0.02	+	-	+	+	+	+	-	+
O161	*Paenibacillus*	KR181835	*P. borealis* KK19	99.3	B03156	0.00	0.00	-	-	+	+	+	+	+	+

[1] Letter in name of isolate indicates the isolation source: L – litter, and O - organic soil

[2] Identification was carried out with EzTaxon on the basis of 16S sequence data.

[3] OTUs sequences were obtained from previous study (1).

[4] Abundance data were obtained from previous study (1).

[5] Enzymatic activities in MM with cellulose: "+": >0.5 μmol min^{-1} mil^{-1} ;" -": < 0.5 μmol min^{-1} mil^{-1} ; "ng": no bacterial growth under assay conditions; "nt": enzymatic activity not tested.

Reference

1. López-Mondéjar, R., Voříšková, J., Větrovský, T., & Baldrian, P. The bacterial community inhabiting temperate deciduous forests is vertically stratified and undergoes seasonal dynamics. *Soil Biol. Biochem*. **87,** 43-50 (2015).

Supplementary Table S2. Features of the draft genome sequences of *Mucilaginibacter* L294, *Pedobacter* O48 and *Luteibacter* L214 isolated from forest topsoil.

Characteristic	*Mucilaginibacter* L294	*Pedobacter* O48	*Luteibacter* L214
Genome size (bp)	5 261 202	6 379 750	4 988 864
Predicted coding sequences	4698	5399	4415
Subsystems	334	355	380
Glycosyl hydrolases (GH)	161	191	57
Polysaccharide lyases (PL)	10	1	1
Carbohydrate esterases (CE)	64	76	47
Cellulose binding modules (CBM)	40	65	21
Auxilliary activity(AA)	13	6	7
Glycosyl transferases (GT)	77	77	42

Supplementary Table S3. Genome content and protein expression of carbohydrate-active enzymes of *Mucilaginibacter* L294, *Pedobacter* O48 and *Luteibacter* L214 isolated from forest topsoil. G – genes predicted in genome sequences, CE – proteins produced during growth on cellulose, ST – proteins produced during growth on wheat straw.

**GH family**	***Mucilagibacter* L294**			***Pedobacter* O48**			***Luteibacter* L214**		
	**G**	**CE**	**ST**	**G**	**CE**	**ST**	**G**	**CE**	**ST**
GH1	1	0	0	1	0	0	0	0	0
GH2	6	0	0	9	1	1	3	0	0
GH3	7	4	0	3	2	2	3	0	0
GH4	0	0	0	0	0	0	1	0	0
GH5	9	3	0	6	3	2	0	0	0
GH9	2	1	0	1	0	0	0	0	0
GH10	1	1	0	1	0	0	0	0	0
GH13	4	3	0	2	0	0	8	2	2
GH15	0	0	0	1	0	0	4	0	0
GH16	5	0	0	1	0	0	0	0	0
GH17	0	0	0	0	0	0	1	0	0
GH18	7	0	0	4	2	2	1	0	0
GH19	1	1	0	0	0	0	2	1	0
GH20	10	3	0	11	2	4	1	1	1
GH23	2	0	0	2	0	0	6	1	0
GH25	1	0	0	1	0	0	0	0	0
GH26	3	0	0	4	1	1	0	0	0
GH27	0	0	0	1	1	1	1	0	0
GH28	2	0	0	4	0	0	0	0	0
GH29	5	3	0	13	1	1	1	0	0
GH30	3	1	0	2	1	1	1	0	0
GH31	6	2	0	4	1	1	0	0	0
GH32	1	0	0	5	1	1	0	0	0
GH33	1	0	0	3	0	2	0	0	0
GH35	1	1	0	0	0	0	2	0	0
GH36	2	2	0	2	1	1	1	0	1
GH37	1	0	0	0	0	0	0	0	0
GH38	1	0	0	0	0	0	0	0	0
GH39	1	0	0	1	0	1	0	0	0
GH42	1	1	0	0	0	0	0	0	0
GH43	11	0	0	10	3	2	1	0	1
GH51	2	0	0	1	0	0	0	0	0
GH63	1	0	0	0	0	0	0	0	0
GH64	0	0	0	0	0	0	1	0	0
GH65	2	1	0	0	0	0	0	0	0
GH73	2	0	0	3	0	0	0	0	0
GH74	4	0	0	1	1	1	0	0	0
GH76	2	0	0	2	0	0	1	0	0
GH77	0	0	0	0	0	0	1	1	1
GH78	9	1	0	14	1	1	0	0	0
GH79	1	0	0	0	0	0	0	0	0
GH88	1	0	0	1	0	0	0	0	0
GH89	0	0	0	1	0	0	0	0	0

GH92	9	3	0	13	2	4	4	1	2
GH93	0	0	0	1	0	0	0	0	0
GH94	0	0	0	0	0	0	1	0	0
GH95	3	0	0	4	1	2	1	0	0
GH97	2	0	0	4	0	0	0	0	0
GH99	4	0	0	7	0	0	3	0	0
GH100	0	0	0	1	0	0	0	0	0
GH103	0	0	0	0	0	0	1	0	0
GH106	3	0	0	6	0	0	0	0	0
GH108	0	0	0	0	0	0	1	0	0
GH109	14	7	0	25	6	7	5	1	0
GH116	0	0	0	3	0	0	0	0	0
GH117	0	0	0	3	0	0	0	0	0
GH125	1	1	0	1	1	1	1	0	0
GH127	2	1	0	3	0	0	0	0	0
GH128	0	0	0	1	0	0	0	0	0
GH130	4	2	0	4	1	0	0	0	0
CBM5	1	0	0	0	0	0	1	0	0
CBM6	0	0	0	5	4	4	0	0	0
CBM8	1	0	0	1	1	1	0	0	0
CBM9	4	0	0	2	0	0	1	1	1
CBM13	2	0	0	2	0	0	0	0	0
CBM21	1	0	0	0	0	0	0	0	0
CBM22	0	0	0	0	0	0	1	0	0
CBM27	0	0	0	0	0	0	0	0	0
CBM30	2	1	0	0	0	0	0	0	0
CBM32	7	4	0	17	2	3	5	1	1
CBM34	1	0	0	0	0	0	0	0	0
CBM35	0	0	0	0	0	0	1	0	0
CBM37	0	0	0	2	1	1	0	0	0
CBM38	0	0	0	1	0	0	0	0	0
CBM40	0	0	0	5	2	1	0	0	0
CBM44	5	1	0	5	3	3	1	0	0
CBM48	1	1	0	1	0	0	3	1	1
CBM50	3	1	0	5	1	1	5	2	1
CBM56	1	0	0	0	0	0	0	0	0
CBM66	5	4	0	11	4	4	3	0	0
CBM67	6	2	0	8	0	2	0	0	0
AA2	1	1	0	1	1	1	1	1	1
AA3	8	2	0	3	1	1	3	0	0
AA4	1	0	0	1	0	0	1	0	0
AA6	3	1	0	1	1	1	2	1	1
CE1	17	7	0	24	8	9	18	5	6
CE2	2	0	0	1	0	0	0	0	0
CE3	15	4	0	10	3	5	4	0	0
CE4	6	1	0	5	0	0	2	0	0
CE5	0	0	0	1	1	1	1	0	0
CE6	2	0	0	4	2	2	0	0	0
CE7	2	0	0	5	1	1	0	0	0
CE8	0	0	0	0	0	0	0	0	0

CE9	2	1	0	3	0	0	0	0	0
CE10	11	5	0	12	4	4	17	5	5
CE11	1	1	0	1	1	0	1	0	1
CE12	1	0	0	3	0	0	0	0	0
CE14	6	3	0	4	2	1	3	0	0
CE15	0	0	0	3	0	0	0	0	0
CE16	0	0	0	0	0	0	1	0	0
PL1	0	0	0	1	0	0	0	0	0
PL5	2	0	0	0	0	0	0	0	0
PL6	1	0	0	0	0	0	0	0	0
PL9	1	0	0	0	0	0	0	0	0
PL17	1	0	0	0	0	0	0	0	0
PL20	1	0	0	0	0	0	0	0	0
PL22	4	0	0	0	0	0	1	1	1
GT1	1	0	0	4	0	0	1	0	0
GT2	29	2	0	22	1	0	12	0	0
GT4	20	1	0	24	1	0	11	0	0
GT5	2	1	0	2	1	0	0	0	0
GT7	0	0	0	2	0	0	0	0	0
GT8	1	0	0	0	0	0	0	0	0
GT9	2	0	0	1	0	0	2	0	0
GT13	0	0	0	3	1	1	0	0	0
GT19	2	1	0	1	0	0	1	0	0
GT20	0	0	0	1	1	0	1	1	1
GT21	1	0	0	1	0	0	0	0	0
GT22	0	0	0	1	0	0	0	0	0
GT26	2	1	0	2	1	1	1	0	0
GT27	1	0	0	0	0	0	1	0	0
GT28	2	0	0	1	0	0	2	0	0
GT30	1	0	0	1	0	0	1	0	0
GT32	0	0	0	2	0	0	0	0	0
GT39	1	0	0	0	0	0	0	0	0
GT46	0	0	0	1	0	0	0	0	0
GT51	5	0	0	4	1	1	3	0	1
GT56	0	0	0	1	0	1	0	0	0
GT59	0	0	0	0	0	0	1	0	0
GT83	4	0	0	2	0	0	4	0	0
GT84	0	0	0	0	0	0	1	0	0
GT87	3	0	0	0	0	0	0	0	0
GT94	0	0	0	1	0	0	0	0	0

Supplementary Figure S1. Screening of potential cellulolytic bacterial strains isolated from forest topsoil. A) Bacterial colonies producing cellulases during the growth in the CMC media produce halos after Congo Red staining. B) Production of extracellular enzymes involved in the degradation of carbohydrates during the growth of several isolates in MM medium with cellulose as the sole carbon source.

A

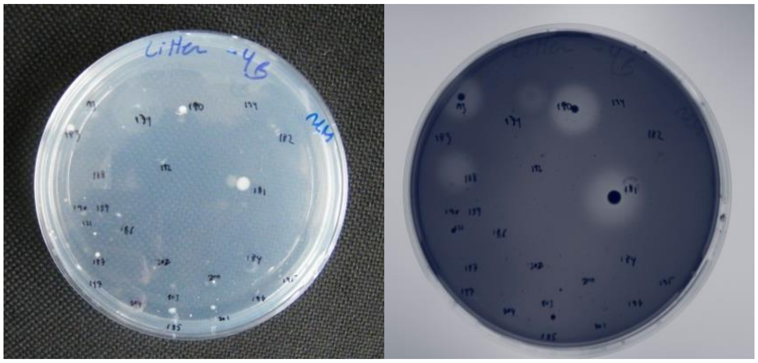

B

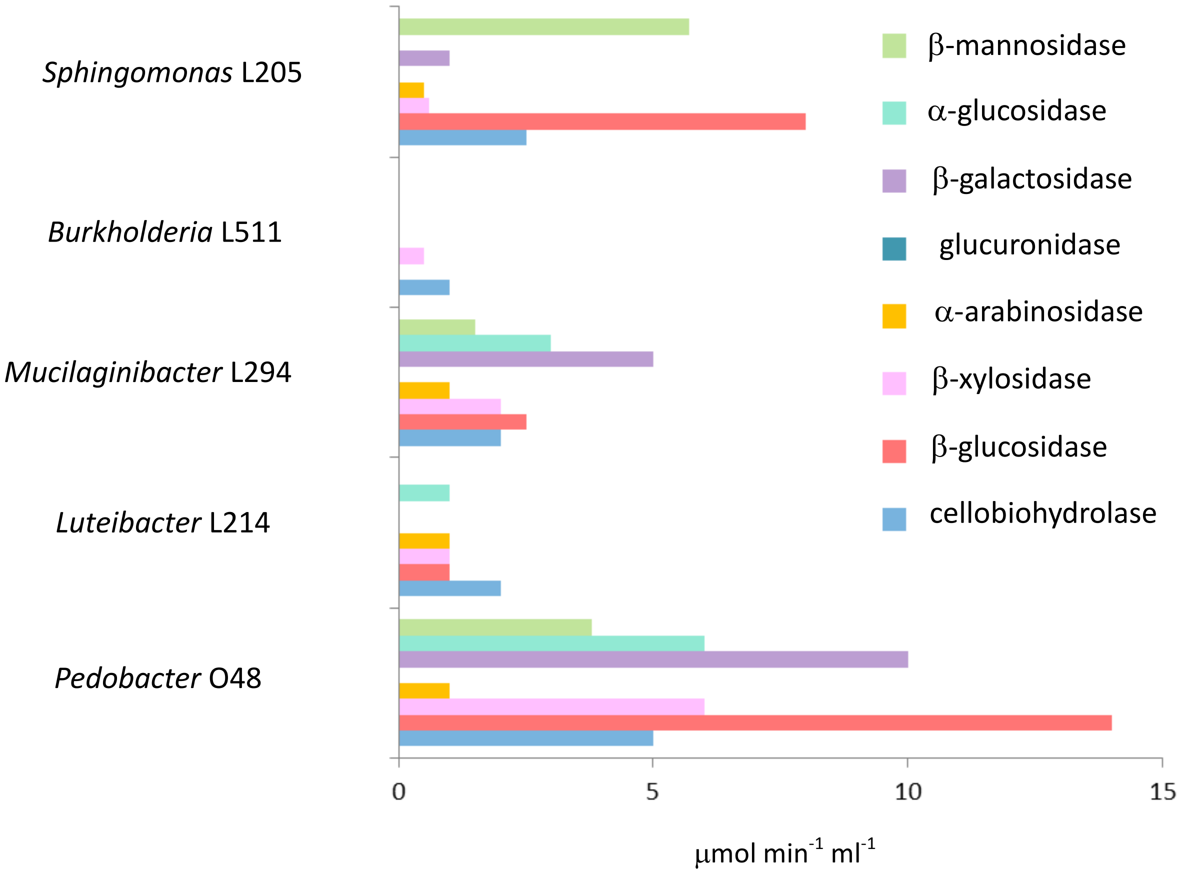

Supplementary Figure S2. Phylogenetic tree of the 16S rRNA gene showing the cellulolytic strains of bacteria isolated from the forest soil organic horizon (O) and litter (L) and their closest identified relatives. The 115 isolates belonged to four major bacterial phyla. The strains representing different OTUs selected for further experiments are marked in red color. Numbers indicate bootstrap support of respective branching points.

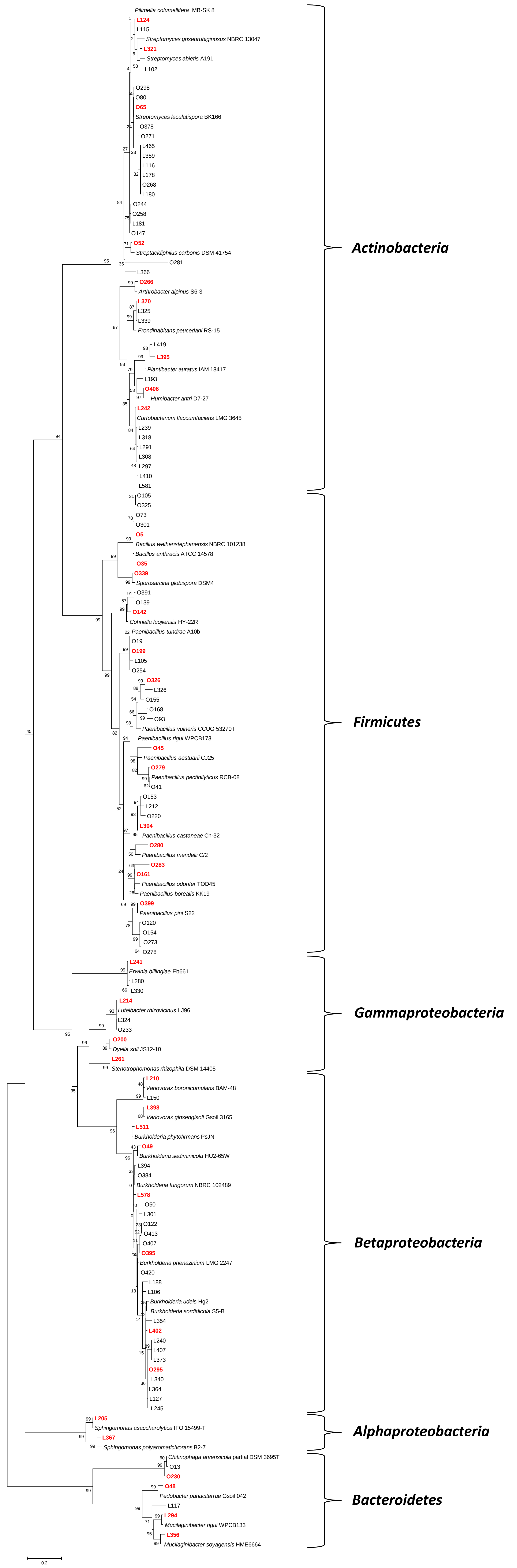

Supplementary Figure S3. Separation of proteins by 1D-SDS-PAGE. A) Proteins from *Mucilaginibacter* L294 cultures growth in cellulose (MC) and straw (MS). B) Proteins from *Pedobacter* O48 cultures growth in cellulose (PEC) and straw (PES). C) Proteins from *Luteibacter* L214 cultures growth in cellulose (LC) and straw (LS). Triplicates were obtained from three independent bacterial cultures. M: marker line.

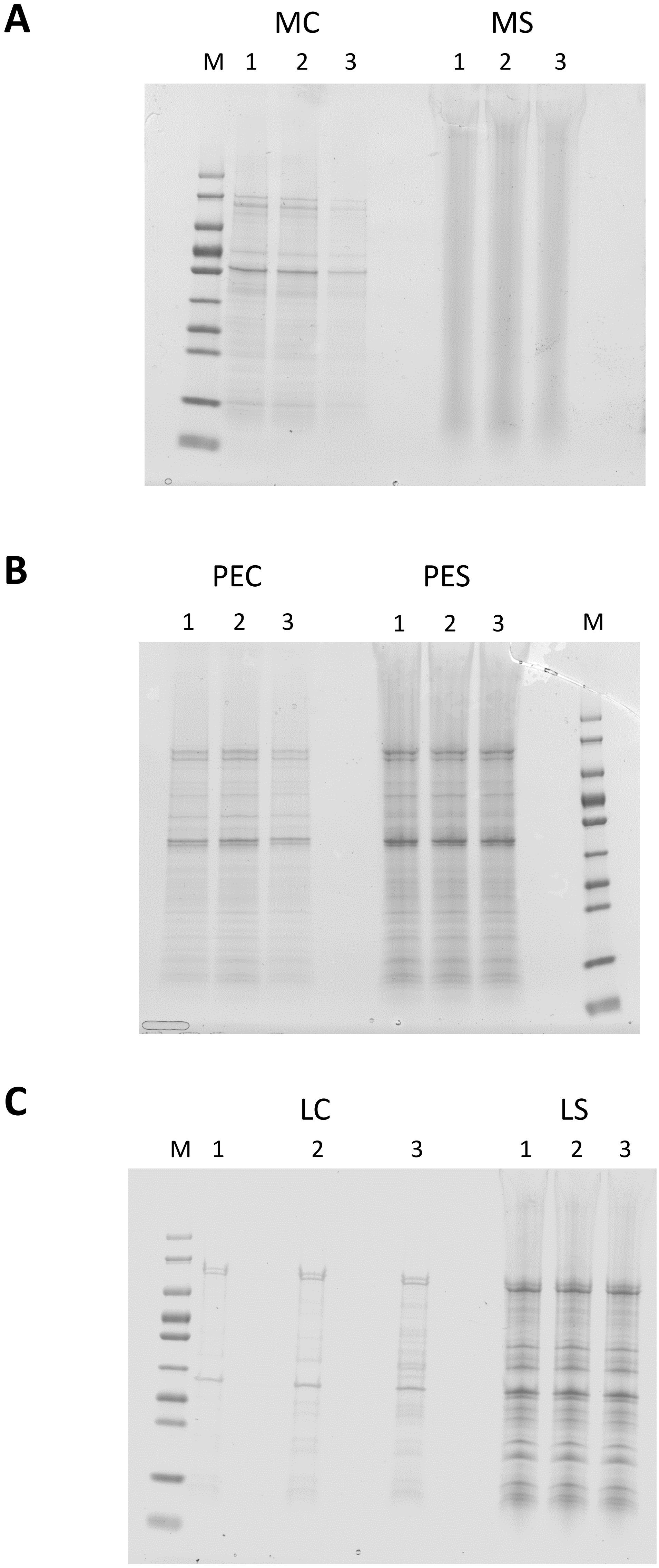

Supplement: Supplementary Information [file srep25279-s1.pdf]
